# Protein arginine methyltransferase 5 (PRMT5) activates WNT/β‐catenin signalling in breast cancer cells via epigenetic silencing of DKK1 and DKK3

**DOI:** 10.1111/jcmm.16260

**Published:** 2021-01-18

**Authors:** Harshita Shailesh, Kodappully S. Siveen, Saïd Sif

**Affiliations:** ^1^ Department of Biological and Environmental Sciences College of Arts and Sciences Qatar University Doha Qatar; ^2^ Flow Cytometry Core Facility Translational Research Institute Hamad Medical Corporation Doha Qatar

**Keywords:** breast cancer, epigenetic silencing, PRMT5, tumour suppressors, WNT/β‐CATENIN proliferative signalling

## Abstract

Protein arginine methyltransferase 5 (PRMT5) activity is dysregulated in many aggressive cancers and its enhanced levels are associated with increased tumour growth and survival. However, the role of PRMT5 in breast cancer remains underexplored. In this study, we show that PRMT5 is overexpressed in breast cancer cell lines, and that it promotes WNT/β‐CATENIN proliferative signalling through epigenetic silencing of pathway antagonists, *DKK1* and *DKK3*, leading to enhanced expression of *c‐MYC*, *CYCLIN D1* and *SURVIVIN*. Through chromatin immunoprecipitation (ChIP) studies, we found that PRMT5 binds to the promoter region of WNT antagonists, *DKK1* and *DKK3*, and induces symmetric methylation of H3R8 and H4R3 histones. Our findings also show that PRMT5 inhibition using a specific small molecule inhibitor, compound 5 (CMP5), reduces PRMT5 recruitment as well as methylation of H3R8 and H4R3 histones in the promoter regions of *DKK1* and *DKK3*, which consequently results in reduced expression *CYCLIN D1* and *SURVIVIN*. Furthermore, CMP5 treatment either alone or in combination with 5‐Azacytidine and Trichostatin A restored expression of *DKK1* and *DKK3* in TNBCs. PRMT5 inhibition also altered the growth characteristics of breast cancer cells and induced their death. Collectively, these results show that PRMT5 controls breast cancer cell growth through epigenetic silencing of WNT/β‐CATENIN pathway antagonists, DKK1 and DKK3, resulting in up‐regulation of WNT/β‐CATENIN proliferative signalling.

## 1 INTRODUCTION

Breast cancer is a major global health issue accounting for 30% of new cases and 14% of deaths among all types of cancer diagnosed every year.^1^ Despite recent progress in treatment and early detection methods, breast cancer remains the most frequently diagnosed cancer in women.^2^ Heterogeneity in the clinical, morphological and molecular nature of the disease presents a major challenge in the selection of adequate treatment strategies. Triple‐negative breast cancers (TNBCs) being the most aggressive type, accounts for 15%‐20% of all breast cancers with poor prognosis and reduced survival rate. TNBC is characterized by the absence of receptors for oestrogen (ER), progesterone (PR) as well as human epidermal growth factor receptor 2 (HER2/neu), which renders it hard to treat, and results in dismal clinical outcomes.^3^ Currently, chemotherapy, alone or in combination with surgery and/or radiotherapy, is the only available option for treating TNBC; however, these approaches are associated with adverse side effects and lack of efficacy. Hence, investigations focusing on discovering novel biomarkers that can be targeted therapeutically in TNBC are needed.

In the last two decades, studies have clearly demonstrated that a combination of genetic lesions and epigenetic alteration of tumour‐suppressor genes and oncogenes contributes to breast carcinogenesis.^4^, ^5^ Aberrant epigenetic events resulting in altered post‐translational modification of histone and non‐histone proteins, DNA methylation and dysregulated miRNA expression play a major role in breast cancer aetiology.^4^, ^5^ For example, elevated occupancy of DNA methyltransferase (DNMT) 1 and histone deacetylase (HDAC) in the promoter region of oestrogen receptor leads to hypermethylation of CpG clusters, resulting in oestrogen receptor gene inactivation in breast cancer cells.^6^ Reduced expression of tumour suppressor miR200b due to DNMT3A‐mediated promoter DNA hypermethylation promotes epithelial to mesenchymal transition and mammosphere formation in TNBCs.^7^ As epigenetic modifications are reversible, targeting enzymes that modulate DNA methylation, and methylation and or acetylation of histone and non‐histone proteins has become a promising therapeutic strategy.

PRMT5 is an important epigenetic modifying enzyme that catalyses monomethylation and symmetric methylation of arginine residues in both histone and non‐histone proteins.^8^, ^9^ Symmetric methylation of specific arginine residues of various proteins by PRMT5 is considered as an essential step in biological processes such as DNA replication and repair, gene transcription, Golgi apparatus and ribosome biogenesis, protein biosynthesis and mRNA splicing.^9^, ^10^ Furthermore, extensive studies have demonstrated that PRMT5 is involved in cell‐cycle control, cell migration and cell reprogramming.^10^ Recent studies have shown that elevated levels of PRMT5 are associated with several cancers including mantle cell lymphoma,^11^, ^12^ metastatic melanoma,^13^ epithelial ovarian cancer,^14^ neuroblastoma,^15^ germ cell tumours,^16^ glioma^17^, ^18^ and colorectal cancers.^19^ PRMT5 in association with BRG1‐ and hBRM‐based SWI/SNF chromatin remodelling complexes induces H3R8 and H4R3 arginine symmetric methylation of promoter histones, which in turn leads to transcriptional repression of target tumour suppressor genes such as Suppressor of Tumorigenicity 7 (*ST7*) and Nonmetastatic 23 (*NM23*).^20^ In addition to these tumour suppressor genes, PRMT5 also reduces transcription of RB family tumour suppressor genes including *RB1*, *RBL1* and *RBL2* in transformed B‐cell lymphocytic leukaemia cell lines.^21^ Furthermore, PRMT5 indirectly down‐regulates the RB1/RBL2‐E2F pathway by enhancing expression of *CYCLIN D1* and promoting inactivation of RB1 and RBL1 through CYCLIN D1‐CDK4/6 dependent phosphorylation.^22^

The role played by PRMT5 in breast carcinogenesis remains underexplored. A prior study by Scoumanne et al. (2009) demonstrated that PRMT5 regulates proliferation of MCF7 cells, and that its knockdown inhibits their proliferation by inducing G1 cell‐cycle arrest, indicating that PRMT5 is a key regulator of cell‐cycle progression.^23^ PRMT5 was also shown to associate with Programmed Cell Death Protein 4 (PDCD4) and reduce its tumour‐suppressor activity in MCF7 cells. Moreover, patients overexpressing both PRMT5 and PDCD4 show poor survival rate compared with those expressing high PDCD4 levels and low levels of PRMT5.^24^ In another study by Yang et al. (2015), PRMT5 levels were found to be up‐regulated in various breast cancer cells including MCF7, MDA‐MB‐231, MCF‐10A and clinical samples of ductal carcinoma, and that its expression is positively associated with enhanced mortality.^25^ More recently, PRMT5 expression was shown to be increased in breast cancer stem cells (BCSCs), and that its knock down reduces proliferation and self‐renewal of BCSCs both in vitro and in vivo.^26^ The mechanism by which PRMT5 regulates breast cancer stem cell function involves up‐regulation of *FOXP1*. PRMT5 binds to the *FOXP1* promoter and induces symmetrical methylation of histone H3R2, which in turn promotes recruitment of the WDR5 subunit of the SET1/MLL methyltransferase complex that is known to methylate H3K4me3, resulting in elevated expression of *FOXP1*. These results indicate that PRMT5 plays an important role in maintaining breast cancer stemness.^26^

Several reports have shown that dysregulation of WNT signalling is tightly linked to carcinogenesis.^27^, ^28^ WNT signalling is activated by binding of WNT ligand to Frizzled receptor and co‐receptor, low‐density lipoprotein receptor‐related protein 5/6 (LRP5/6). Binding of WNT to its cognate receptor inhibits formation of the cytosolic destruction complex, which is composed of AXIN1, AXIN2, protein‐phosphatase‐2A (PP2A), GSK3β, casein kinase1 (CK1) and adenomatous polyposis coli (APC). Consequently, cytosolic β‐CATENIN levels increase, which then translocates to the nucleus, interacts with T‐cell factor and lymphoid enhancer factor (TCF/LEF), and activates transcription of target genes such as *c‐MYC*, *CYCLIN D1* and *SURVIVIN*.^29^, ^30^, ^31^, ^32^ WNT/β‐CATENIN signalling is known to be activated in 50% of breast cancer patients,^33^ and promoter DNA hypermethylation of pathway antagonists such as *APC*, *DKK3*, *SFRP1* and *SFRP2* has been reported in many breast cancer samples.^34^, ^35^, ^36^

We have recently shown that PRMT5 activates WNT/β‐CATENIN signalling pathway in three different types of non‐Hodgkin’s lymphoma cell lines, mouse primary lymphoma cell lines and clinical samples through epigenetic silencing of *AXIN2* and *WIF1*.^37^ However, the role played by PRMT5 in regulating WNT/β‐CATENIN signalling in breast cancer remains unknown. Therefore, we have investigated in the current study the impact of PRMT5 inhibition on WNT/β‐CATENIN signalling in triple‐negative breast cancer (TNBC) cells. Our findings indicate that elevated levels of PRMT5 promote WNT/β‐CATENIN proliferative signalling through transcriptional repression of WNT antagonists, *DKK1* and *DKK3*. ChIP assay confirmed binding of PRMT5 to the promoter region of WNT antagonists, *DKK1* and *DKK3*, and hypermethylation of promoter H3R8 and H4R3. Moreover, PRMT5 inhibition either alone or in combination with Trichostatin A (TSA) and 5‐Azacytidine (5‐Aza) led to transcriptional derepression of *DKK1* and *DKK3*, and decreased expression of WNT/β‐CATENIN target genes, *CYCLIN D1* and *SURVIVIN*. These changes were also accompanied by reduced proliferation, migration, and invasion and enhanced cell death of breast cancer cells.

## 2 MATERIALS AND METHODS

### 2.1 Cell culture

Human breast cancer cell lines HCC1937 and BT549 cells were cultured in RPMI 1640 medium (Gibco, Life Technologies Inc.) supplemented with 10% foetal bovine serum (FBS). BT549 cells were supplemented with insulin (0.023 U/mL). MCF7 cells were cultured in DMEM medium (Gibco, Life Technologies Inc.) supplemented with 10% FBS. Human mammary epithelial cells (HMECs) were grown in mammary epithelial cell basal medium (American type culture collection) supplemented with rH‐insulin (5 μg/mL), L‐Glutamine (6 mmol/L), Epinephrine (1 μmol/L), Apo‐transferrin (5 μg/mL), rH‐TGF‐α (5 ng/mL), pituitary extract (0.4%) and hydrocortisone (100 ng/mL).

### 2.2 Real‐Time PCR

Total RNA was extracted using TRIzol reagent (Invitrogen^TM^). Briefly, TNBC cells from a T75 flask were removed using 750 μL of TRIzol reagent, transferred to an eppendorf tube and incubated at room temperature for 5 minutes. Next, 300 μL of chloroform was added and samples were mixed vigorously and incubated at room temperature for 10 minutes. Samples were then centrifuged at 4°C for 15 minutes before the upper layer containing RNA was transferred to a new eppendorf tube containing 500 μL of isopropanol. After incubation at room temperature for 10 minutes, the reactions were centrifuged at 4°C for 15 minutes. The RNA pellet was rinsed with 75% ethanol, air‐dried before resuspension in 20 μL of nuclease‐free water. Next, mRNA was converted into cDNA using High‐Capacity cDNA Reverse Transcription Kit as per manufacturer’s instructions (Applied Biosystems^TM^). Briefly, 2 μg of total RNA was reverse transcribed in a 20‐μL reaction mixture containing 2.5 μmol/L random primers, 100 mmol/L dNTP Mix and Taqman reverse transcription reagents. To measure the mRNA levels of WNT target genes and antagonists, real‐time PCR was carried out using TaqMan^TM^ Universal PCR Master Mix in a 20‐μL reaction as described previously.^11^ The following primers and probe sets were used to detect *PRMT5* (forward, 5*'*‐CCAGAGCCTTGGAAGCA‐3*'* and reverse, 5*'*‐CTGATGGGCAAGGGGAAT‐3*'*, probe#68), *CYCLIN D1* (forward, 5′‐GAAGATCGTCGCCACCT‐3′ and reverse, 5′‐GACCTCCTCCTCGCACTT‐3′, probe#67), *c‐MYC* (forward, 5′‐ACCAGCTGGAGATGGTGA‐3′ and reverse, 5′‐CGGGTCGCAGATGAAACT‐3′, probe#52), *SURVIVIN* (forward, 5*'‐*GCCCAGTGTTTCTTCTG‐3*'* and reverse, 5*'*‐CCGGACGAATGCTTTTT‐3*'*, probe#11), *AXIN1* (forward, 5*'*‐AGCTCTCCGAGACAGAGA‐3*'* and reverse, 5*'*‐CAACGATGCTGTCACACG‐3*'*, probe#6), *AXIN2* (forward, 5*'*‐CTAGGAGTGCGTTCATGG‐3*'* and reverse, 5*'*‐GGGACGTAGTGCAAAGC‐3*'*, probe#6), *WIF1* (forward, 5*'*‐AAGCCAGCCTCATACATG‐3*'* and reverse, 5'‐AAGTGAAGGCGTGTGCT‐3*'*, probe#40), *DKK1* (forward, 5*'*‐CAGGCGTGCAAATCTGTCT‐3*'* and reverse, 5*'*‐GATTTTGATCAGAAGACACAC‐3*'*, probe#4), *DKK2* (forward, 5*'*‐GGCAGTAAGAAGGGCA‐3*'* and reverse, 5*'*‐CCTCCCAACTTCACACTC‐3*'*, probe#43), *DKK3* (forward, 5*'*‐CACATCTGTGGGAGACGAA‐3*'* and reverse, 5*'*‐CCCACAGTCCTCGTCGAT‐3*'*, probe#29), *DKK4* (forward, 5*'*‐AGGAGGTGCCAGCGAGA‐3*'* and reverse, 5*'*‐ CATCTTCCATCGTAGTACA‐3*'*, probe#37), *SFRP2* (forward, 5*'*‐TAGCAGCGACCACCTC‐3*'* and reverse, 5'‐GCAGGCTTCACATACC‐3*'*, probe#83), *SFRP3* (forward, 5*'*‐GATCCAAGGAAGCGGTGA‐3*'* and reverse, 5*'*‐GTGGACACAAGGATCTGG‐3*'*, probe#52), *SFRP4* (forward, 5*'*‐CCTGAAGCCATCGTCAC‐3*'* and reverse, 5*'*‐ATCATGTCTGGTGTGATGT‐3*'*, probe#88), *SFRP5* (forward, 5*'*‐CTCAGGGTCTCAGAAAG‐3*'* and reverse, 5*'*‐GAGCCCCTCCACCTTT‐3*'*, probe#52), and *β‐ACTIN* (forward, 5*'*‐AGCTACGAGCTGCCTGAC‐3*'* and reverse, 5*'*‐GGCTGGAAGAGTGCCTCA‐3*'*, probe#9). *β‐ACTIN* was used as internal control to normalize expression of tested genes.

### 2.3 Western blot analysis

Whole‐cell extracts were prepared in radioimmune precipitation assay (RIPA) buffer (50 mmol/L Tris‐HCl [pH 7.5], 150 mmol/L NaCl, 1% NP‐40, 0.1% SDS, 0.5% sodium deoxycholate, 0.5 mmol/L DTT, 0.5 mmol/L PMSF and 2.5 mmol/L Roche protease inhibitor cocktail). The extracts were subjected to Western blot analysis as described previously.^11^ Briefly, 20 to 40 μg of total protein were separated using 7‐12% SDS‐PAGE and transferred to PVDF membrane. The membrane containing transferred proteins was blocked by incubating with 5% BSA containing 0.05% Tween‐20 and incubated overnight at 4°C with primary antibody to detect CYCLIN D1 (Abcam, ab134175), c‐MYC (Abcam, ab62928), SURVIVIN (Abcam, ab76424), α‐TUBULIN (Abcam, ab4074), DKK1 (Abcam, ab109416), DKK3 (Abcam, ab186409), β‐ACTIN (Cell Signaling Technology, 4970), CYCLIN D3 (Cell Signaling Technology, DCS22) and PRMT5 (Thermo Fisher, MA1‐25470). After incubation with primary antibody, the membrane was treated with HRP‐conjugated goat anti‐mouse (Amersham Biosciences, NA931) or anti‐rabbit (Amersham Biosciences, NA934V) secondary antibody. Next, proteins were visualized using the ECL detection kit (Amersham, RPN2209) in a Western blot imager (Flurochem E system, proteinsimple).

### 2.4 Chromatin immunoprecipitation (ChIP) assay

Chromatin immunoprecipitation was carried out as described previously.^11^ Cross‐linked chromatin was resuspended in ChIP lysis buffer (100 mmol/L Tris‐HCl [pH 8.6], 15 mmol/L NaCl, 60 mmol/L KCl, 1 mmol/L CaCl_2_, 3 mmol/L MgCl_2_) supplemented with protease inhibitors, Aprotinin (10 μg/mL), PMSF (100 mmol/L), Pepstatin (2.25 μg/mL), and Leupeptin (10 μg/mL), and fragmented using Q55 sonicator (Qsonica). Sonicated chromatin was further digested by micrococcal nuclease (MNase) (0.6 Units) treatment at 37°C for 20 minutes. MNase‐treated chromatin was analysed by agarose gel electrophoresis to ensure that DNA fragment sizes did not exceed 500 bp. To evaluate PRMT5 recruitment as well as PRMT5‐induced H3R8 and H4R3 symmetric methylation marks, chromatin was immunoprecipitated overnight at 4°C using either pre‐immune or immune antibodies against PRMT5, H3(Me_2_)R8 and H4(Me_2_)R3 in the presence of protein A‐Sepharose beads, which were pre‐blocked with sheared salmon sperm DNA (0.2 mg/mL) and BSA (0.5 mg/mL). The retained complexes were washed successively with mixed micelle buffer (20 mmol/L Tris‐HCl [pH 8.1], 50 mmol/L NaCl, 5 mmol/L EDTA, 5% w/v sucrose, 0.2% Triton X‐100, 0.2% SDS), buffer 250 (50 mmol/L HEPES [pH 7.5], 0.1% sodium deoxycholate, 250 mmol/L NaCl, 1 mmol/L EDTA, 0.2% Triton X‐100), and wash buffer (10 mmol/L Tris‐HCl [pH 8], 150 mmol/L LiCl, 1 mmol/L EDTA, 0.5% sodium deoxycholate, 0.25% NP‐40). Next, chromatin was eluted with 200 μL of elution buffer (50 mmol/L Tris‐HCl [pH 8.0], 10 mmol/L EDTA, 1% SDS) at 65°C for 10 minutes, and cross‐links were reversed by incubating the samples at 65°C overnight. Chromatin was then incubated with proteinase K (5 μg/mL), yeast tRNA (0.5 μg/mL) and TE [pH 7.6] at 37°C for 2 hours before 50 μL of 4 M LiCl was added, and DNA was purified by phenol and chloroform extraction. Purified DNA was resuspended in 30 μL of Tris‐EDTA (pH 8.0) and 3 μL of RNase A (10 mg/mL). To amplify promoter sequences of PRMT5 target genes, 6 μL of eluted DNA was used in a 20‐μL real‐time PCR reaction mixture containing the following primer pairs and specific Roche Applied Science universal probes (*DKK1* forward, 5*'*‐CCGGATAATTCAACCCTTAC‐3*'* and reverse, 5*'*‐GCAGACGACTTTAATAAATGC‐3*'*, probe#18; *DKK3* forward, 5*'*‐AGGAGCTGCAAGTGCGTTAT‐3*'* and reverse, 5*'*‐GGCCTTAGTCTGCCGTGAT‐3*'*, probe#19; *RBL2* forward, 5*'*‐TCCAGGCGATGAGGAAGT‐3*'* and reverse, 5*'*‐TGACGTGTGAAGGTTTCG‐3*'* probe#64).

### 2.5 Transwell migration and invasion assays

Boyden migration assay was carried out using a 24‐well plate with polycarbonate transwell inserts of pore size 8.0 μm (BD, Falcon). Boyden invasion assay was conducted using the BD Bio‐Coat Matrigel system. Approximately 3 × 10^4^ cells resuspended in 500 μL serum‐free medium with or without PRMT5 inhibitor were added into the upper well of Boyden chamber. The lower chamber was filled with 1.3 mL of culture medium containing serum for 24 or 48 hours. After incubation, cells that migrated to the lower surface of the upper chamber were fixed by treating them first with 4% formaldehyde, followed by absolute methanol, and then cells were stained with 1% Crystal violet. Images from 4 random fields were captured using an inverted microscope, and cells were counted using ImageJ software.

### 2.6 Proliferation assay

Approximately 2 × 10^4^ cells were seeded into a 24‐well plate, and 24 hours later, cells were treated with or without PRMT5 inhibitor for different time intervals. Next, cells were collected, resuspended in 20‐μL solution containing equal volume of culture medium and Trypan blue dye, and counted in order to determine the number of viable cells.

### 2.7 Flow cytometry

Approximately 6 × 10^4^ cells were seeded in a 6‐well plate prior to treatment with or without inhibitor for 24 or 48 hours. Next, cells were harvested, washed and resuspended in 1× Annexin binding buffer containing Annexin V‐FITC and PI, and then incubated for 15 minutes in the dark at room temperature. Stained cells were analysed with BD LSRFortessa flow cytometer using FACS Diva software (Becton Dickinson).

### 2.8 ELISA Assay

Levels of secreted antagonists of the WNT/β‐CATENIN pathway were detected by ELISA technique using DuoSet ELISA kit (R&D system). Essentially 100 μL of capture antibody was added to 96‐well culture plate and incubated at room temperature overnight. Next, the antibody solution was removed, and wells were washed twice with 400 μL of wash buffer (0.005% Tween‐20 in filter‐sterilized 1× PBS). Wells were blocked by incubating with 300 μL of blocking buffer (1% BSA in filter‐sterilized 1× PBS) for 1 hour at room temperature. Blocked wells were washed twice with wash buffer before adding 100 μL of either specific standard protein or sample solution. Wells were sealed with adhesive plastic strips, incubated at room temperature for 2 hours before washing twice with wash buffer. Next, 100 μL of Streptavidin‐HRP A was added to each well and incubated in the dark for 20 minutes at room temperature. Wells were then washed, and 100 μL of Substrate solution containing 1:1 mixture of colour reagent A (H_2_O_2_) and colour reagent B (Tetramethylbenzidine) was added before incubating at room temperature in the dark for 20 minutes. After incubation, 50 μL of stop solution (2N H_2_SO_4_) was added, and the optical density of each well was read at 450 nm (TECAN, Infinite 200PRO).

### 2.9 Statistical analysis

The real‐time RT‐PCR and ChIP experiments were performed at least two times using different biological replicates, and the data obtained were represented as mean ± SD. Statistical validation of the data obtained from multiple samples within different groups was performed by two‐way ANOVA analysis using GraphPad Prism 7 software, and *P* values ≤.05 were considered as statistically significant.

## 3 RESULTS

### 3.1 PRMT5 levels and WNT/β‐CATENIN target genes are up‐regulated in breast cancer cell lines

**FIGURE 1:**
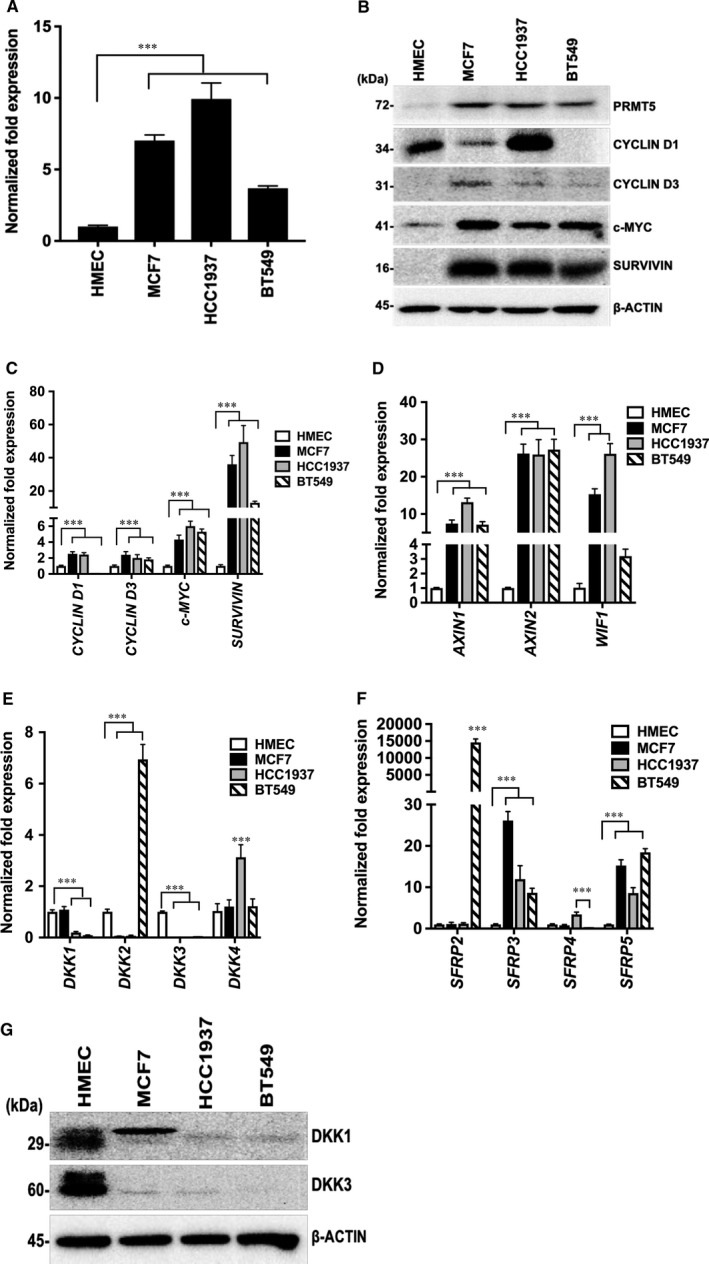
Expression of PRMT5 and WNT/‐CATENIN target genes is elevated and that of WNT/‐CATENIN antagonists, DKK1 and DKK3, is down‐regulated in breast cancer cells. mRNA levels of PRMT5 (A), WNT/‐CATENIN target genes including *CYCLIN D1*, *CYCLIN D3*, *c‐MYC* and *SURVIVIN* (C) WNT/‐CATENIN antagonists *AXIN1*, *AXIN2*, and *WIF1* (D), *DKK1‐4* (E) and *SFRP2‐5* (F) in normal HMECs or MCF7, HCC1937 and BT549 cells were detected by real‐time RT‐PCR using 2 µg of total RNA. The experiment was performed twice with three technical replicates, and *‐ACTIN* was used as internal control. The results are represented as mean ± SD. RIPA extracts (20 µg) from either normal HMECs or breast cancer cells were analysed by SDS‐PAGE to detect PRMT5, CYCLIN D1, CYCLIN D3, c‐MYC and SURVIVIN (B) as well as WNT/‐CATENIN antagonists, DKK1 and DKK3 (G) by Western blotting. ‐ACTIN was used as loading control. *** indicates *P* values < 10^−3^

Previous studies have shown that PRMT5 levels are elevated in a wide variety of cancer cells including glioblastoma, melanoma, non‐small cell lung carcinoma, lymphoma and leukaemia cells.^11^, ^13^, ^18^, ^38^ Enhanced PRMT5 expression has also been shown in breast cancer cell lines as well as clinical samples of ductal carcinoma.^25^ In light of these findings, we investigated PRMT5 expression in HER2‐negative MCF7 cells, and TNBC cell lines, HCC1937 and BT549 (Figure 1). Real‐time RT‐PCR revealed that PRMT5 mRNA levels were enhanced by sevenfold (*P* < 10^−3^) in MCF7, 9.9‐fold (*P* < 10^−3^) in HCC1937 and 3.7‐fold (*P* < 10^−3^) in BT549 cells compared with normal human mammary epithelial cells (HMECs) (Figure 1A). In accord with this result, Western blot analysis showed that PRMT5 protein expression was increased in breast cancer cell lines compared to normal HMECs (Figure 1B). These results show that PRMT5 levels are up‐regulated in transformed breast epithelial cells.

Our recent work using three different types of aggressive lymphoma cell lines has shown that PRMT5 promotes WNT/β‐CATENIN proliferative signalling by transcriptionally repressing expression of pathway antagonists, *AXIN2* and *WIF1*.^37^ Consequently, expression of WNT/β‐CATENIN target genes such as *CYCLIN D1*, *c‐MYC* and *SURVIVIN* increases.^37^ To investigate whether elevated levels of PRMT5 regulate WNT/β‐CATENIN proliferative signalling in breast cancer, we checked expression of all three WNT/β‐CATENIN pathway target genes in MCF7, HCC1937 and BT549 cell lines. Analysis of mRNA expression showed that *CYCLIN D1* was increased by 2.5‐fold (*P* < 10^−3^) in MCF7, and 2.4‐fold (*P* < 10^−3^) in HCC1937 compared with control HMECs (Figure 1C). In agreement with work by Lin et al. (2000), which showed previously that *CYCLIN D1* is not expressed in BT549 cells, we were unable to detect *CYCLIN D1* mRNA in BT549 cells (Figure 1C).^33^ An initial study by Bartkova et al. (1998) investigated the abundance of D‐type CYCLINs in human diploid cells and tumour cell lines and showed that CYCLIN D3 is the most widely expressed D‐type CYCLIN in human diploid cells and cancer cell lines including BT549 cells.^39^ Therefore, we measured *CYCLIN D3* mRNA levels in transformed breast epithelial cells. We found that *CYCLIN D3* mRNA levels were increased by 2.4‐fold (*P* < 10^−3^) in MCF7, twofold (*P* < 10^−3^) in HCC1937 cells and 1.8‐fold (*P* < 10^−3^) in BT549 cells (Figure 1C). Furthermore, we found that *c‐MYC* mRNA levels were elevated by 4.3‐ to sixfold (*P* < 10^−3^) in all three tested cell lines. Similarly, *SURVIVIN* mRNA was enhanced by 12.9‐ to 49.3‐fold (*P* < 10^−3^) in MCF7, HCC1937 and BT549 cell lines compared with normal HMECs (Figure 1C).

Having found that mRNA levels of WNT/β‐CATENIN target genes are increased in transformed breast cell lines, we checked c‐MYC, SURVIVIN, and CYCLIN D1 and 3 protein levels. Western blot analysis showed that CYCLIN D1 expression is significantly up‐regulated in HCC1937, compared to MCF7 and BT549 where its expression was either reduced or not detected, respectively. In contrast, CYCLIN D3 protein levels were increased in all three types of breast cancer cells compared to normal HMECs. In addition, both c‐MYC and SURVIVIN protein expression was elevated in all three breast cancer cell lines (Figure 1B). Collectively, these results show that expression of PRMT5 and WNT/β‐CATENIN target genes is enhanced in MCF7, HCC1937 and BT549 cells.

We have recently shown that PRMT5 promotes WNT/β‐CATENIN proliferative signalling through transcriptional repression of pathway antagonists, AXIN2 and WIF1, in three different types of lymphoma cells.^37^ These findings prompted us to investigate the role of PRMT5 in WNT/β‐CATENIN signalling by examining expression of pathway antagonists including AXIN1, AXIN2, WIF1, DKK1‐4 and SFRP1‐5 in breast cancer cell lines. In contrast to our results in the different lymphoma cell types, we found that *AXIN1*, *AXIN2* and *WIF1* mRNA levels were elevated in transformed breast epithelial cells (Figure 1D). *AXIN1* mRNA levels were increased by 7.1‐ to 13.2‐fold (*P* < 10^−3^), while *AXIN2* mRNA levels were augmented by 25.9‐ to 27.3‐fold, (*P* < 10^−3^) in MCF7, HCC1937 and BT549 cells compared with normal HMECs. Similarly, *WIF1* also showed increased mRNA levels in MCF7 cells (15.3‐fold, *P* < 10^−3^), HCC1937 (26.1‐fold, *P* < 10^−3^) and BT549 cells (3.2‐fold, *P* = .075) compared to normal HMECs (Figure 1D).

Next, we examined the mRNA levels of the Dickkopf gene family members, DKK1‐4, and we found that *DKK1* mRNA levels were unaffected in MCF7 cells; however, they were substantially decreased in HCC1937 (fivefold, *P* < 10^−3^) and BT549 cells (11.1‐fold, *P* < 10^−3^) (Figure 1E). *DKK2* mRNA levels were reduced in MCF7 and HCC1937 cells by 21.3‐fold (*P* < 10^−3^) and 21.6‐fold (*P* < 10^−3^), respectively. In contrast, *DKK2* mRNA levels were elevated by 6.9‐fold (*P* < 10^−3^) in BT549. *DKK3* mRNA expression was undetectable in MCF7 and HCC1937 cells and was reduced by 38.6‐fold (*P* < 10^−3^) in BT549 cells. Expression of *DKK4* mRNA was unaffected in MCF7 and BT549 cells compared with normal HMECs and was up‐regulated by 2.4‐fold (*P* < 10^−3^) in HCC1937 cells (Figure 1E).

When we examined expression of secreted frizzled‐related proteins (SFRPs), we found that *SFRP2* mRNA was unaffected in MCF7 and HCC1937 cells compared with control HMECs (Figure 1F). This was not the case in BT549 cells, which showed elevated levels of *SFRP2* mRNA (14 609‐fold, *P* < 10^−3^). Two other members of the SFRP family, *SFRP3* and *SFRP5*, showed substantial increase (8.6‐ to 26.1‐fold, *P* < 10^−3^) in their mRNA levels in breast cancer cells compared with normal HMECs. Expression of *SFRP4* mRNA in MCF7 cells did not differ from normal HMECs; however, its level was elevated in HCC1937 (3.4‐fold, *P* < 10^−3^) and reduced in BT549 cells (6.5‐fold, *P* < 10^−3^) (Figure 1F).

As both *DKK1* and *DKK3* showed significant reduction in their mRNA levels in both TNBCs, HCC1937 and BT549, we evaluated their protein levels in both normal and transformed breast cells. In accord with our RT‐PCR results, Western blot analysis showed that both DKK1 and DKK3 proteins were suppressed in HCC1937 and BT549 compared with normal HMECs (Figure 1G). In contrast to TNBCs, MCF7 cells had detectable expression of DKK1 protein, which showed altered mobility by SDS‐PAGE compared with normal HMECs. In addition, similar to TNBCs, DKK3 protein was significantly suppressed in MCF7 cells compared with normal HMECs (Figure 1G). Collectively, these results indicate that expression of WNT/β‐CATENIN pathway antagonists, DKK1 and DKK3, is altered in HCC1937 and BT549 cell lines, and suggest that PRMT5 might be involved in their regulation.

### 3.2 PRMT5 epigenetically silences DKK1 and DKK3, and reduces their expression

In light of our recent work, which showed that PRMT5 epigenetically represses transcription of WNT/β‐CATENIN antagonists *AXIN1*, *AXIN2* and *WIF1* by directly binding to their promoter region and inducing H3R8 and H4R3 symmetric methylation,^37^ we wanted to verify whether PRMT5 is involved in transcriptional repression of WNT/β‐CATENIN antagonists, *DKK1* and *DKK3* in breast cancer cells. To analyse PRMT5 recruitment and enrichment of its induced epigenetic marks in the promoter region of *DKK1* and *DKK3*, chromatin immunoprecipitation (ChIP) experiments were carried out. Cross‐linked chromatin from MCF7, HCC1937 and BT549 cells was immunoprecipitated with either pre‐immune, anti‐PRMT5, anti‐H3(Me_2_)R8 or anti‐H4(Me_2_)R3 antibodies, which were previously characterized and shown to be highly specific.transcriptional repression of pathway antagonists

**FIGURE 2:**
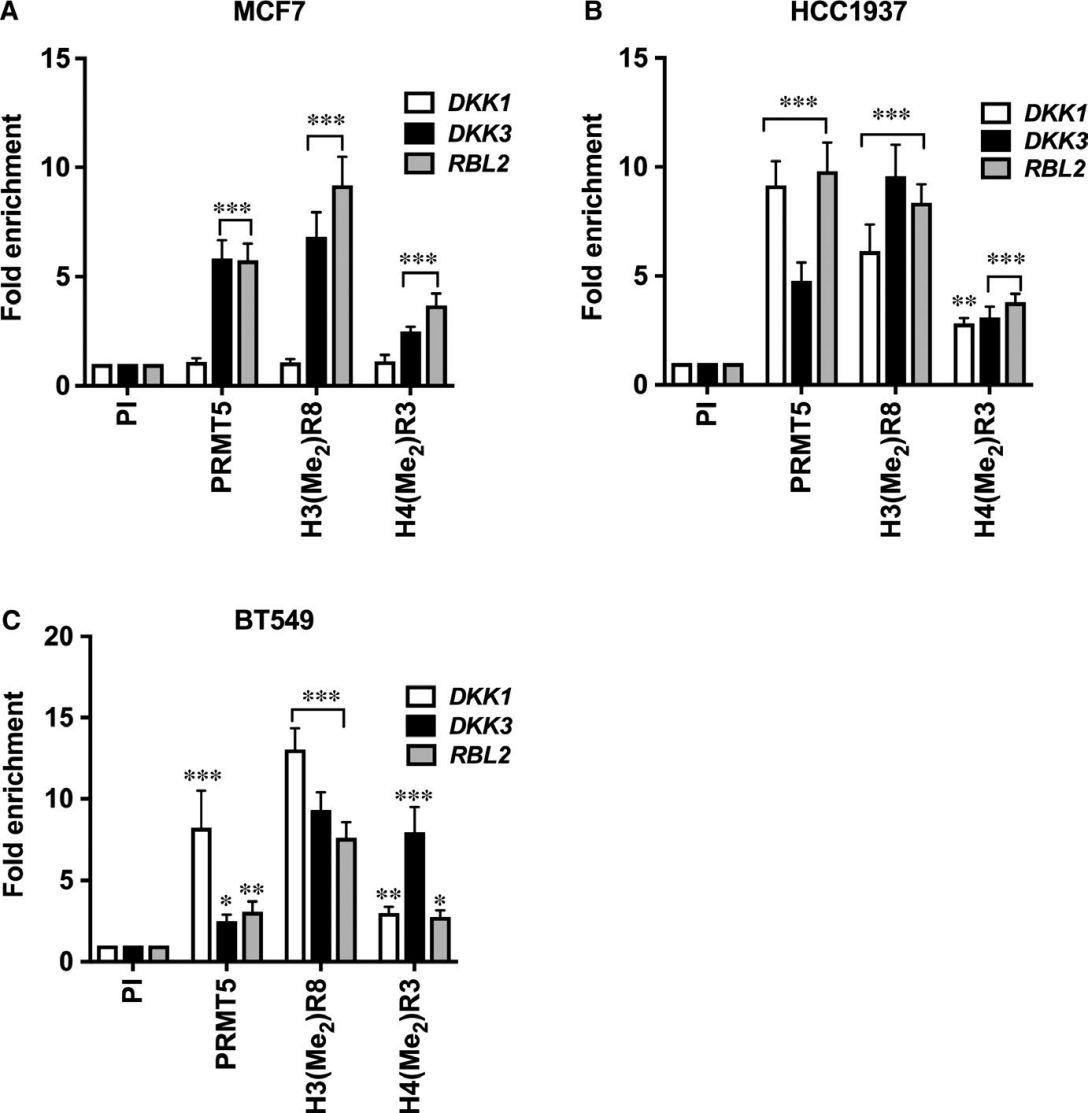
PRMT5 epigenetically suppresses expression of WNT/‐CATENIN antagonists, *DKK1* and *DKK3* in breast cancer cells. ChIP assays were performed using cross‐linked chromatin from either MCF7 (A), HCC1937 (B) or BT549 (C) cells. Immunoprecipitation of nucleoprotein complexes was performed using pre‐immune (PI), anti‐PRMT5, anti‐H3(Me_2_)R8 or anti‐H4(Me_2_)R3 antibodies, and the purified DNA was used to detect the promoter sequences of *DKK1* and *DKK3* by real‐time PCR using specific primers and probe sets. ChIP assays were repeated two times with three technical replicates. The results are represented as mean ± SD. *** indicates *P* values < 10^−3^, ** indicates *P* values < 10^−2^ and * indicates *P* value < 10^−1^

Real‐time PCR amplification of the immunoprecipitated DNA showed that there was no significant enrichment in PRMT5 recruitment or H3(Me_2_)R8 and H4(Me_2_)R3 epigenetic marks at the *DKK1* promoter in MCF7 cells (Figure 2A). In contrast, there was a 5.8‐fold (*P* < 10^−3^) increase in PRMT5 binding to the promoter region of *DKK3*, which was accompanied by enhanced symmetric methylation of H3R8 (6.8‐fold, *P* < 10^−3^) and H4R3 (2.5‐fold, *P* < 10^−3^) in MCF7 cells (Figure 2A).

ChIP assay in HCC1937 cells showed enhanced PRMT5 recruitment (9.2‐fold, *P* < 10^−3^) and elevated methylation of H3R8 (6.1‐fold, *P* < 10^−3^) and H4R3 (2.8‐fold, *P* = .001) at the *DKK1* promoter (Figure 2B). Similarly, increased PRMT5 recruitment and enrichment of its epigenetic marks (3.1‐ to 9.6‐fold, (*P* < 10^−3^) were observed in the promoter region of *DKK3* (Figure 2B). Enrichment of PRMT5 recruitment in the promoter region of *DKK1* was enhanced by 8.2‐fold (*P* < 10^−3^), whereas PRMT5 binding to the promoter region of *DKK3* was increased by 2.5‐fold (*P* = .038) in BT549 cells (Figure 2C). Consistent with these results, elevated levels of H3(Me_2_)R8 (13.1‐fold, *P* < 10^−3^) and H4(Me_2_)R3 (threefold, *P* = .003) were detected in the promoter region of *DKK1*. Furthermore, symmetric methylation of H3R8 (9.3‐fold, *P* < 10^−3^) and H4R3 (eightfold, *P* < 10^−3^) was also detected in the promoter region of *DKK3* (Figure 2C). These results indicate that PRMT5 regulates WNT/β‐CATENIN proliferative signalling through epigenetic transcriptional repression of pathway antagonists, *DKK1* and *DKK3*, in HCC1937 and BT549 cells.

### 3.3 Inhibition of PRMT5 either alone or in combination with HDACs and DNMT3A derepresses DKK1 and DKK3 in breast cancer cells

**FIGURE 3:**
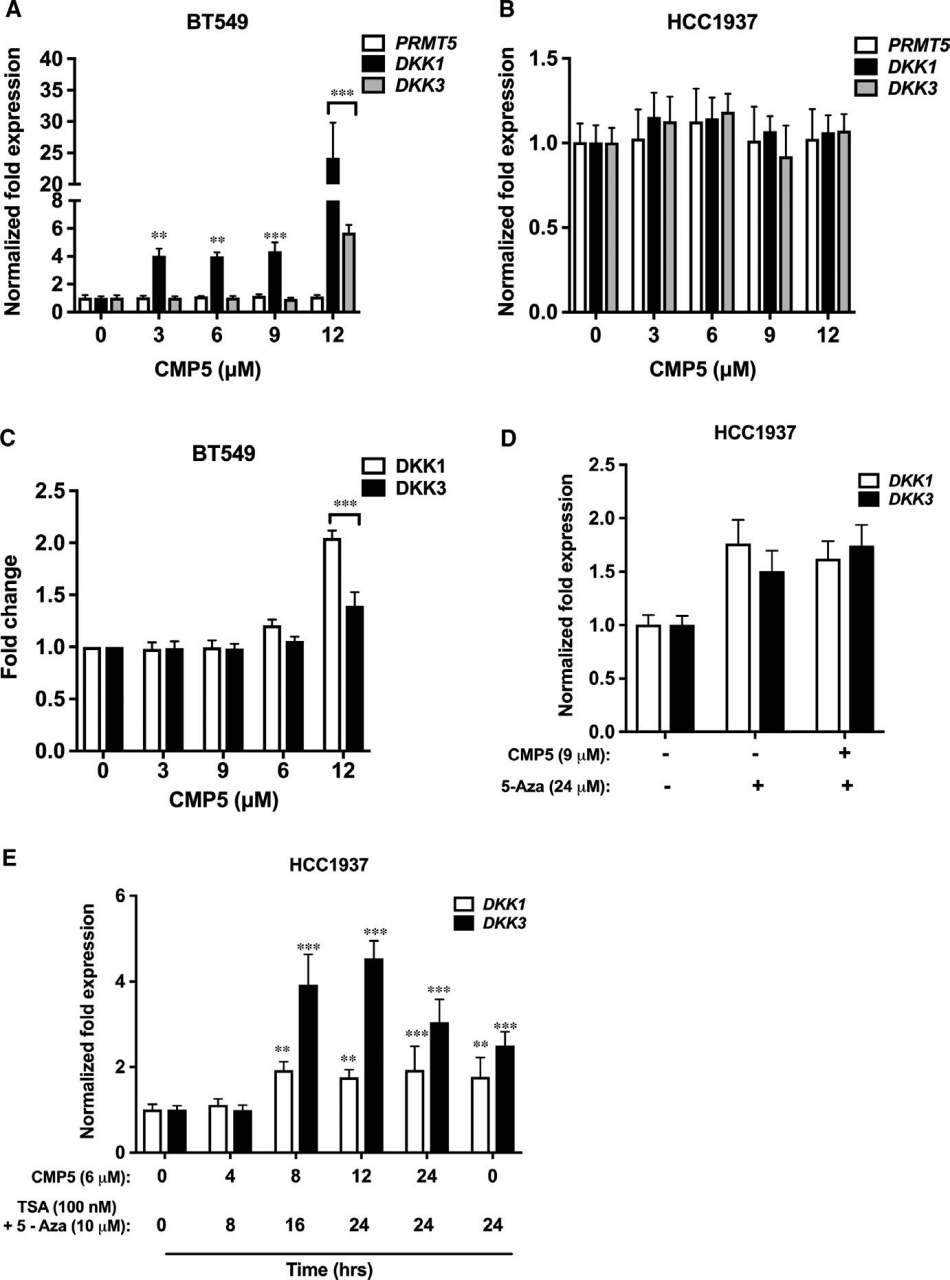
PRMT5 inhibition induces transcriptional derepression of WNT/‐CATENIN antagonists *DKK1* and *DKK3* in TNBC cells. mRNA levels of *DKK1* and *DKK3* were analysed by real‐time RT‐PCR using 2μg of total RNA extracted from BT549 (A) and HCC1937 (B) treated with increasing amounts of CMP5 (0, 3, 6, 9, 12 μmol/L) for 48 h and 24 h, respectively. (C) Protein levels of DKK1 and DKK3 were analysed by ELISA after collecting the culture media from either DMSO‐ or CMP5‐treated BT549 cells for 48 h. (D) mRNA levels of *DKK1* and *DKK3* were analysed by real‐time RT‐PCR using 2μg of total RNA extracted from HCC1937 cells treated with 5‐Azacytidine (24 μmol/L) alone or in combination with CMP5 (9 μmol/L) for 24 h. (E) mRNA levels of DKK1 and DKK3 were analysed by real‐time RT‐PCR using 2 μg of total RNA extracted from HCC1937 cells treated with suboptimal concentration of CMP5 (6 μmol/L) in combination with TSA (100 nmol/L) and 5‐Azacytidine (10 μmol/L) for the indicated time intervals. All the experiments were repeated two times with three technical replicates. The results are represented as mean ± SD. *** indicates *P* values < 10^−3^ and ** indicates *P* values < 10^−2^

In light of our findings, which show that PRMT5 protein levels are inversely correlated with WNT/β‐CATENIN antagonists, we wanted to evaluate the effect of PRMT5 inhibition on expression of *DKK1* and *DKK3*, as well as WNT/β‐CATENIN target genes, *c‐MYC*, *CYCLIN D1* and *SURVIVIN* in TNBCs. HCC1937 and BT549 cells were treated with increasing concentrations of CMP5 for 24 or 48 hours, respectively, and total RNA was analysed. Real‐time RT‐PCR data indicated that transcription of *DKK1* was induced fourfold to 4.3‐fold (*P* < 10^−2^) at lower concentrations of CMP5 (3 μmol/L to 9 μmol/L) and was significantly up‐regulated by 24‐fold (*P* < 10^−3^) at 12 μmol/L in BT549 cells (Figure 3A). Similarly, *DKK3* mRNA levels were up‐regulated by 5.6‐fold (*P* < 10^−3^) in the presence of 12 μmol/L of CMP5 in BT549 cells (Figure 3A). The mRNA levels of *DKK1* and *DKK3* genes were unaffected in HCC1937 cells (Figure 3B), suggesting that there might be other mechanisms involved in their regulation. As control, the mRNA levels of PRMT5 were also measured and were found to be unaffected in the presence of CMP5 (Figure 3A,B). To determine the biological significance of *DKK1* and *DKK3* transcriptional derepression, we measured the levels of DKK1 and DKK3 proteins in BT549 cells treated with CMP5 (Figure 3C). ELISA analysis showed that both DKK1 and DKK3 were derepressed by twofold and 1.4‐fold (*P* < 10^−3^), respectively (Figure 3C). Consistent with the real‐time RT‐PCR results, levels of DKK1 and DKK3 proteins were unchanged in HCC1937 cells (Data not shown).

As PRMT5 inhibition could not restore expression of *DKK1* and *DKK3* in HCC1937 cells, we investigated other epigenetic mechanisms that could be involved in their transcriptional regulation. PRMT5 has previously been shown to work in concert with DNA methyltransferase 3A (DNMT3A).^40^, ^41^ Therefore, we reasoned that PRMT5 might be promoting DNA methylation of *DKK1* and *DKK3* promoters in HCC1937 cells. To address this question, we treated HCC1937 cells with the DNA methyltransferase inhibitor, 5‐Azacytidine (5‐Aza). HCC1937 cells were incubated with increasing amounts of 5‐Aza for either 24, 48 or 72 hours, and viability was determined by Trypan blue dye exclusion assay, which showed that even a higher concentration of 5‐Aza (24 μmol/L) does not have significant cytotoxicity on HCC1937 cells (data not shown). Next, we examined the effect of 5‐Aza (24 μmol/L) alone or in combination with suboptimal amount of CMP5 (9 μmol/L), on transcriptional derepression of *DKK1* and *DKK3* in HCC1937 cells. Treatment of HCC1937 cells with 24 μmol/L of 5‐Azacytidine for 48 hours did not impact transcription of *DKK1* (1.75‐fold, *P* < 10^−3^) and *DKK3* (1.5‐fold, *P* < 10^−3^). Furthermore, sequential incubation of HCC1937 cells with 24 μmol/L of 5‐Aza first for 24 hours, followed by addition of 9 μmol/L CMP5 for another 24 hours also did not induce significant transcriptional derepression of *DKK1* (1.6‐fold, *P* < 10^−3^) and *DKK3* (1.7‐fold, *P* < 10^−3^) (Figure 3D).

Deacetylation of histone lysine residues by histone deacetylase enzymes (HDAC) has been shown to be essential for transcriptional silencing of genes.^42^ An earlier study by our group showed that PRMT5 can associate with mSin3A/histone deacetylase 2 (HDAC2) to form a transcriptional repression complex with the BRG1‐based hSWI/SNF chromatin remodelers.^20^ In addition, we have previously shown that PRMT5 associates with an NF‐κB transcriptional repressor complex containing HDAC3, which promotes deacetylation of H3K14 lysine, and triggers gene silencing in lymphoma cells.^12^ To investigate whether histone deacetylation is involved in transcriptional repression of WNT antagonists, *DKK1* and *DKK3*, we treated HCC1937 cells with Trichostatin A (TSA). Real‐time RT‐PCR analysis showed that treatment with either 100 nmol/L or 300 nmol/L of TSA for 24 hours had no effect on *DKK1* and *DKK3* transcription in HCC1937 cells. Similarly, combinatorial treatment with 9 µmol/L of CMP5 and either 100 or 300 nmol/L of TSA for 24 hours also failed to induce transcriptional activation of WNT antagonists, *DKK1* and *DKK3* in these cells (data not shown).

Previous studies have shown that simultaneous inhibition of histone deacetylase enzyme and DNA methyltransferase enzyme results in restoration of epigenetically silenced genes.^43^, ^44^ Therefore, we investigated the effect of combinatorial treatment of TSA and 5‐Aza in inducing transcriptional derepression of *DKK1* and *DKK3* in HCC1937 cells. We incubated HCC1937 cells with a combination of suboptimal doses of TSA (100 nmol/L) and 5‐Aza (10 μmol/L) for 24 hours (Figure 3E). Real‐time RT‐PCR data showed that this treatment increased mRNA level of *DKK1* and *DKK3* by 1.8‐fold (*P *= .006) and 2.5‐fold (*P* < 10^−3^), respectively (Figure 3E). As histone lysine deacetylation, histone arginine methylation and DNA methylation can all work together to induce gene silencing, we hypothesized that combinatorial treatment with inhibitors against HDAC, PRMT5 and DNMT3A might enhance transcriptional derepression of *DKK1* and *DKK3* in HCC1937 cells as compared to treatment with CMP5 in the presence of either TSA or 5‐Aza. First, we treated HCC1937 cells with 100 nmol/L of TSA and 10 μmol/L of 5‐Aza for 24 hours, followed by incubation with 9 µmol/L of CMP5 for an additional 24 hours. The results of this study showed that mRNA level of *DKK1* and *DKK3* by 1.6‐fold and twofold, respectively (data not shown), suggesting that combinational treatment with all three inhibitors was not able to induce further expression of *DKK1* and *DKK3* in these cells.

As PRMT5‐induced H4R3 histone methylation is known to direct binding of DNMT3A, and induce gene silencing,^41^ we tested the effect of an initial treatment with CMP5, followed by addition of TSA and 5‐Aza in HCC1937 cells. We treated HCC1937 cells with 6 µmol/L of CMP5 for 4 hours, and then added 100 nmol/L of TSA and 10 µmol/L of 5‐Aza for an additional 8 hours. Results from this experiment showed that *DKK1* and *DKK3* expression was not affected (Figure 3E). Next, we treated HCC1937 cells with CMP5 (6 µmol/L) for 8 hours, followed by an incubation with TSA (100 nmol/L) and 5‐Aza (10 µmol/L) for another 16 hours. Real‐time RT‐PCR analysis showed that the mRNA levels of *DKK1* were enhanced by 1.9‐fold (*P* = .023) and those of *DKK3* were elevated by 3.9‐fold (*P* < 10^−3^) (Figure 3E). Furthermore, when we increased time of incubation with CMP5 to 12 hours, followed by incubation with TSA and 5‐Aza for an additional 24 hours, *DKK1* transcriptional derepression remained at 1.8‐fold (*P* = .0063), whereas *DKK3* mRNA was derepressed by 4.5‐fold (*P* < 10^−3^) (Figure 3E). Similarly, treating CMP5 for 24 hours and then incubating with TSA and 5‐Aza for next 24 hours resulted in derepression of mRNA levels of *DKK1* and *DKK3* by 1.9‐fold (*P* = .0055) and threefold (*P* < 10^−3^), respectively (Figure 3E). Collectively, these results indicate that PRMT5 is an important epigenetic enzyme, which is involved either alone or in combination with other epigenetic enzymes in silencing WNT pathway antagonists, *DKK1* and *DKK3* in TNBC cells.

### 3.4 Inhibition of PRMT5 down‐regulates WNT/β‐CATENIN target genes in HCC1937 and BT549 cell lines

**FIGURE 4:**
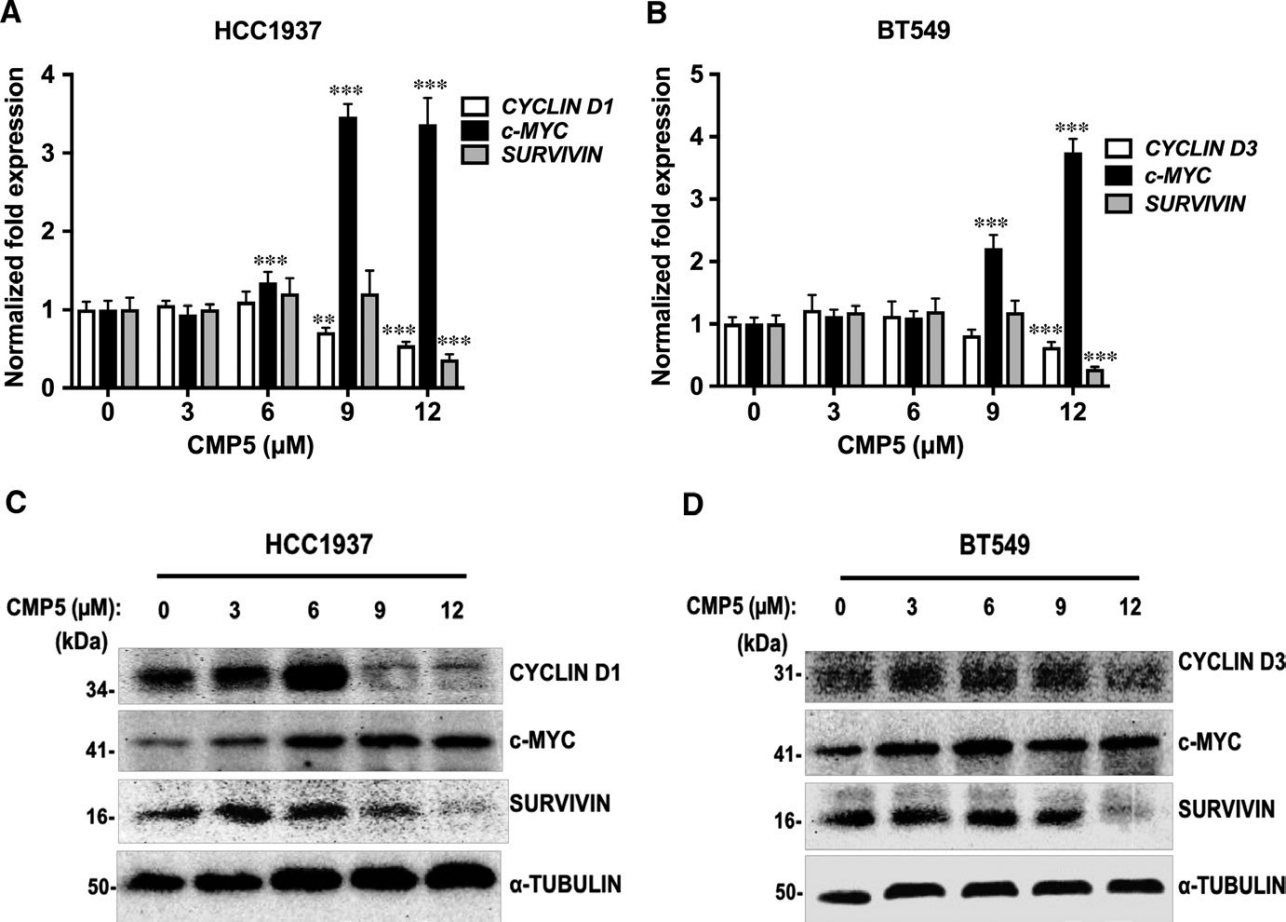
PRMT5 inhibition down‐regulates expression of CYCLIN D1 and SURVIVIN in TNBC cells. HCC1937 (A) and BT549 (B) TNBC cells were treated with increasing amounts of CMP5 (0, 3, 6, 9, 12 μmol/L), and total RNA was extracted 24 h and 48 h post‐treatment, respectively. Levels of WNT/‐CATENIN target genes were analysed by real‐time RT‐PCR using gene‐specific primers and probe sets. The experiment was repeated two times with three technical replicates and *‐ACTIN* was used as an internal control. The data shown in the graph represent the mean for each concentration SD. Approximately 20 µg of RIPA extracts from either treated or non‐treated HCC1937 (C) and BT549 (D) cells were analysed by immunoblotting using the indicated antibodies. ⍺‐TUBULIN was detected to show equal loading. *** indicates *P* values < 10^−3^, and ** indicates *P* value < 10^−2^

Having found that PRMT5 inhibition can lead to transcriptional derepression of *DKK1* and *DKK3*, we wished to evaluate expression of WNT/β‐CATENIN target genes, *c‐MYC*, *CYCLIN D1* and *SURVIVIN*. HCC1937 treatment with 12 μmol/L of CMP5 for 24 hours triggered reduction in mRNA levels of *CYCLIN D1* and *SURVIVIN* by 1.8‐fold (*P* < 10^−3^) and 2.8‐fold (*P* < 10^−3^), respectively (Figure 4A). Similar repression was seen in BT549 cells, which showed reduced expression of *CYCLIN D3* (1.6‐fold, *P* < 10^−3^) and *SURVIVIN* mRNAs (3.6‐fold, *P* < 10^−3^), after 48 hours incubation with 12 μmol/L of CMP5 (Figure 4B). As *c‐MYC* is a known target gene of the WNT signalling pathway, we expected that its mRNA levels would be reduced upon PRMT5 inhibition. Surprisingly, *c‐MYC* mRNA levels were up‐regulated by 3.4‐fold (*P* < 10^−3^) upon treatment of HCC1937 cells with 9 μmol/L and 12 μmol/L of CMP5 for 24 hours (Figure 4A). Similar results were observed with BT549 cells, which showed an up‐regulation of *c‐MYC* mRNA levels by 2.2‐fold (*p* < 10^−3^) and 3.7‐fold (*p* < 10^−3^) treatment with 9 μmol/L and 12 μmol/L of CMP5 for 48 hours, respectively (Figure 4B).

Next, we examined the impact of PRMT5 inhibition on protein expression of WNT/β‐CATENIN target genes in TNBCs. In accord with the RT‐PCR data, CYCLIN D1 and SURVIVIN protein levels decreased in HCC1937 cells after treatment with either 9 or 12 μmol/L of CMP5 for 24 hours (Figure 4C). BT549 cells also showed significant reduction in CYCLIN D3 and SURVIVIN protein expression after 48 hours incubation with 12 μmol/L of CMP5 (Figure 4D). In stark contrast, the levels of c‐MYC protein showed an increase with either 9 or 12 μmol/L of CMP5 treatment in both TNBCs (Figure 4C,D). These results indicate that through its ability to regulate WNT/β‐CATENIN signalling, PRMT5 controls expression of key survival and cell‐cycle regulators including CYCLIN D1, CYCLIN D3 and SURVIVIN in TNBC cells.

### 3.5 PRMT5 inhibition alters its recruitment and H3R8 and H4R3 symmetric methylation in the promoter region of *DKK1* and *DKK3*

**FIGURE 5:**
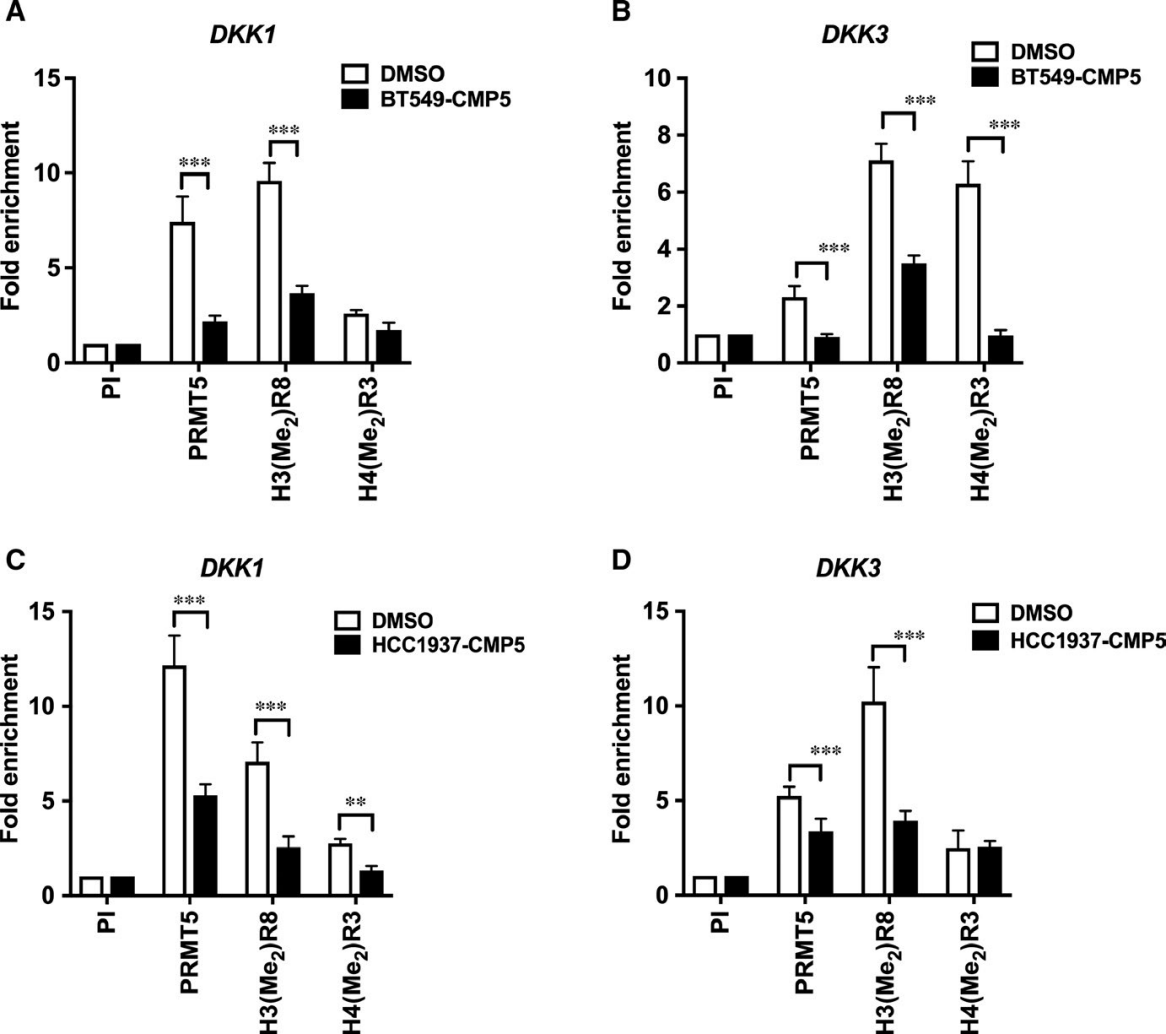
PRMT5 inhibition alters its recruitment and symmetric dimethylation of histones, H3R8 and H4R3 on promoter regions of *DKK1* and *DKK3*. ChIP assay was performed to detect recruitment of PRMT5 and enrichment of its epigenetic marks in the promoter region of WNT/‐CATENIN antagonists as described in Figure 3. Cross‐linked chromatin from either DMSO‐ or CMP5‐treated BT549 (A and B) and HCC1937 (C and D) cells was immunoprecipitated using PI or the indicated immune antibodies. *DKK1* and *DKK3* promoter sequences of were detected by real‐time PCR using specific primers and probe sets. The experiment was repeated two times with three technical replicates, and data in each graph represent the mean ± SD. *** indicates *P* values < 10^−3^, and ** indicates *P* value < 10^−2^

Treatment of BT549 with PRMT5 inhibitor resulted in *DKK1* and *DKK3* transcriptional derepression (Figure 3A). Therefore, we conducted ChIP experiments to monitor PRMT5 recruitment as well as methylation of H3R8 and H4R3 in the promoter region of *DKK1* and *DKK3* in the presence and absence of CMP5 (Figure 5). ChIP analysis revealed that PRMT5 recruitment was enhanced by 7.4‐fold (*P* < 10^−3^) and 2.3‐fold (*P* < 10^−3^) in the promoter regions of *DKK1* and *DKK3*, respectively, in control DMSO‐treated BT549 cells. Consistent with these results, symmetric methylation of H3R8 was enriched by 9.6‐ (*P* < 10^−3^) and 7.1‐fold (*P* < 10^−3^) in the promoter of *DKK1* and *DKK3*, respectively. Similarly, methylation of H4R3 histones in the promoter region of *DKK1* and *DKK3* was increased by 2.6‐ to 6.3‐fold (*P* < 10^−3^). When BT549 cells were treated with CMP5, there was a 3.4‐fold (*P* < 10^−3^) decrease in PRMT5, which was accompanied by a 2.6‐fold (*P* < 10^−3^) and 1.5‐fold (*P* = .072) reduced methylation of H3R8 and H4R3, respectively, in the *DKK1* promoter (Figure 5A,B). In addition, BT549 cells showed that CMP5 treatment resulted in a decrease of PRMT5 binding by 2.6‐fold (*P* < 10^−3^), and H3R8 and H4R3 methylation by twofold, (*P* < 10^−3^) and 6.6‐fold (*P* < 10^−3^), respectively, in the *DKK3* promoter region (Figure 5A,B).

ChIP assay in DMSO‐treated HCC1937 cells showed that PRMT5 recruitment was enriched in the promoter region of *DKK1* and *DKK3* by 12.1‐ (*P* < 10^−3^) and 5.2‐fold (*P* < 10^−3^), respectively. Consistent with this result, symmetric methylation of H3R8 was enhanced by 7.06‐ to 10.2‐fold (*P* < 10^−3^) on both promoters, respectively. Similarly, symmetric methylation of H4R3 was increased by 2.8‐ and 2.5‐fold (*P* < 10^−3^) on *DKK1* and *DKK3* promoters, respectively. Treatment of HCC1937 cells with CMP5 reduced enrichment of PRMT5 by 2.3‐fold (*P* < 10^−3^) and decreased methylation of H3R8 and H4R3 by 2.8‐fold (*P* < 10^−3^) and 2.1‐fold (*P* = .007) at the *DKK1* promoter, respectively (Figure 5C,D). Similarly, treatment with CMP5 reduced PRMT5 binding by 1.6‐fold (*P* < 10^−3^) and symmetric methylation of H3R8 by 2.6‐fold (*P* < 10^‐3^) at the *DKK3* promoter in HCC1937 cells. However, there was no reduction in symmetric methylation of H4R3 at the *DKK3* promoter upon treatment of HCC1937 cells with CMP5 (Figure 5C,D). These results indicate that PRMT5 regulates proliferative WNT/β‐CATENIN signalling through direct epigenetic repression of pathway antagonists, *DKK1* and *DKK3*.

### 3.6 PRMT5 inhibition reduces viability of breast cancer cells

**FIGURE 6:**
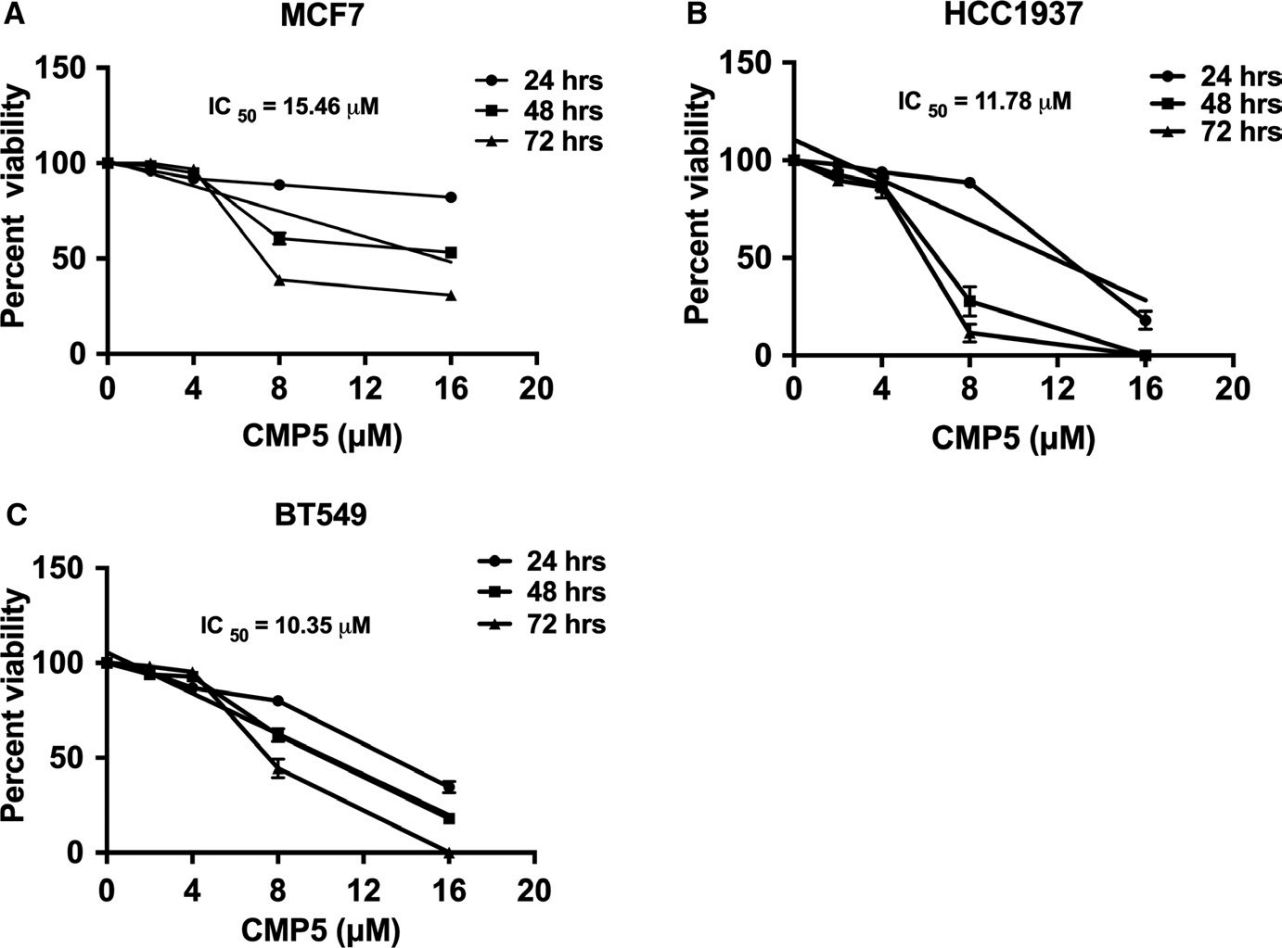
PRMT5 inhibition reduces the viability of breast cancer cells in vitro. MCF7 (A), HCC1937 (B) and BT549 (C) were incubated with different concentrations of CMP5 for 24, 48 and 72 h. Cell viability was measured by Trypan blue dye exclusion assay, and data represented in each graph is from two biological replicates with three technical replicates

To assess the effect of PRMT5 inhibition on viability of MCF7, HCC19 and BT54937 cell lines, we incubated them with increasing amounts of CMP5 (2, 4, 8 and 16 μmol/L) for either 24, 48 or 72 hours (Figure 6). Trypan blue dye exclusion indicated that treatment with lower concentrations of CMP5 (2 and 4 μmol/L) did not reduce breast cancer cell viability significantly at any of the tested time intervals. However, treatment with 8 μmol/L of CMP5 caused a reduction in MCF7 cell viability by 11.4% (*P* < 10^−3^), 39% (*P* < 10^−3^) and 61.2% (*P* < 10^−3^) after 24, 48 and 72 hours, respectively, compared to control DMSO‐treated cells. Treatment with 16 μmol/L of CMP5 showed significant decrease in viability by 18% (*P* < 10^−3^), 46.9% (*P* < 10^−3^) and 69.1% (*P* < 10^−3^) after 24, 48 and 72 hours, respectively. Based on these results, we found that CMP5 had a LC_50_ value of 15.46 µmol/L at 48 hours for MCF7 cells (Figure 6A). Similarly, incubating HCC1937 cells with 8 μmol/L of CMP5 for 24, 48 and 72 hours time intervals reduced cell viability by 11.5% (*P* < 10^−3^), 72.2% (*P* < 10^−3^) and 88.5% (*P* < 10^−3^), respectively. HCC1937 cell viability was substantially reduced by 82% (*P* < 10^−3^) after 24 hours treatment with 16 μmol/L of CMP5, and all cells were dead (0% viability, *P* < 10^−3^) after 48 and 72 hours treatment. Data analysis showed that CMP5 had a LC_50_ of 11.7 μmol/L after 24 hours treatment for HCC1937 cells (Figure 6B). Treating BT549 cells with 8 μmol/L of CMP5 reduced their viability by 20% (*P* < 10^−3^), 38% (*P* < 10^−3^) and 55.6% (*P* < 10^−3^) after 24, 48 and 72 hours post‐treatment, respectively. In addition, treatment with 16 μmol/L of CMP5 reduced viability of BT549 cells by 65.5% (*P* < 10^−3^) and 82% (*P* < 10^−3^) after 24 and 48 hours, respectively. Treatment for 72 hours with 16 μmol/L of CMP5 resulted in complete loss of cell viability. When we plotted the results, we found that CMP5 had a LC_50_ of 10.35 µmol/L at 48 hours for BT549 cells (Figure 6C).

### 3.7 PRMT5 is required for migration and invasion of TNBC cells, and its inhibition induces apoptosis

**FIGURE 7:**
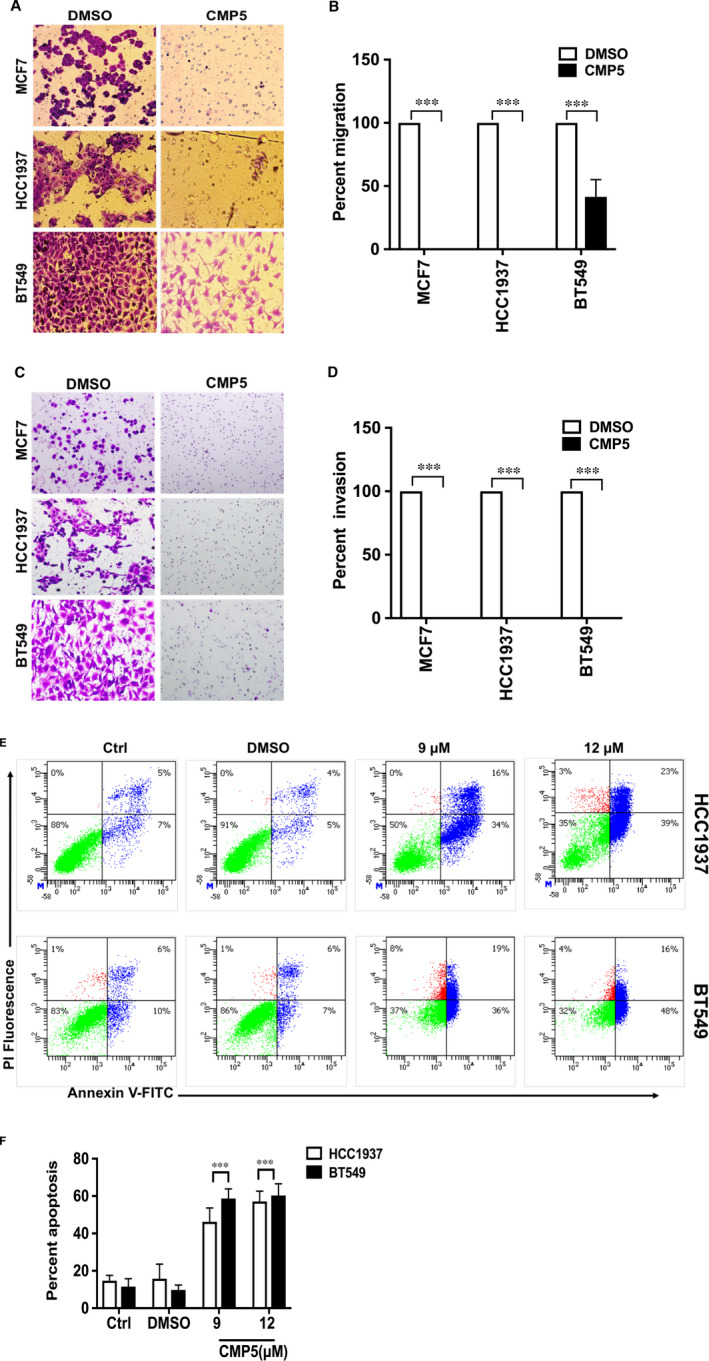
PRMT5 inhibition reduces migration, invasion and induces apoptosis of breast cancer cells. (A) Migration of breast cancer cells was evaluated using Matrigel coated Boyden chamber. Breast cancer cells were treated with either DMSO or CMP5 for 48 h (MCF7 and BT549) or 24 h (HCC1937) as described in Materials and Methods. The migrated cells were stained with crystal violet, and the photomicrographs were captured using an inverted microscope with a magnification of 100×. (B) The percentage of migrated cells was determined using ImageJ software, and the migration capacity was normalized to that of DMSO‐treated cells. (C) Invasion of breast cancer cells was assessed using modified Boyden chamber. MCF7, HCC1937 and BT549 cells were treated with either DMSO or CMP5 for 24 h or 48 h as described in Materials and Methods section. The percentage of invading cells was determined after staining with crystal violet using an inverted microscope with a magnification of 100X. (D) Membrane‐associated invading cells were quantified using ImageJ software, and the per cent invasion was determined in comparison to DMSO‐treated cells. (E) HCC1937 and BT549 cells were treated with different concentrations of CMP5 as indicated, and the number of dead cells was measured by FACS analysis after staining cells with ANNEXIN V and propidium iodide (PI). (F) The average result from the two biological replicates used in E is represented as mean ± SD. All experiments were carried out twice in duplicates, and the data are represented as mean ± SD. *** indicates *P* values < 10^−3^

Enhanced migration is an intrinsic property of cancer cells, which also helps metastasis, a hallmark of TNBC.^45^ The role of PRMT5 in breast cancer cell migration was studied by Boyden chamber assay. Treatment of MCF7, HCC1937 and BT549 with CMP5 at their respective LC_50_ dose, showed that migration of MCF7 and HCC1937 cells was completely inhibited compared to control DMSO‐treated cells. However, migration of BT549 cells was reduced by 58.6% (*P* < 10^−3^) (Figure 7A,B). To assess if PRMT5 inhibition can impact invasiveness of breast cancer cell lines, we utilized Boyden invasion assay. Our findings showed that PRMT5 inhibition using the respective LC_50_ completely abolished invasion by MCF7, HCC1937 and BT549 (Figure 7C,D).

Our findings showed that upon incubation with PRMT5 inhibitor, CMP5, all treated cell lines became less viable (Figure 6). In order to determine how CMP5 reduced viability of TNBC cells, we treated them with 9 µmol/L and 12 µmol/L of CMP5 and analysed ANNEXIN V positive cells using FACS analysis. Treatment of HCC1937 cells with 9 µmol/L and 12 µmol/L of CMP5 for 24 hours, using four technical replicates from two biological replicates, showed an average of 46.2% (*P* < 10^−3^) and 57.1% (*P* < 10^−3^) cell death, respectively (Figure 7E,F). Similarly, incubating BT549 cells with 9 µmol/L and 12 µmol/L of CMP5 for 48 hours resulted in an average of 58.7% (*P* < 10^−3^) and 60.3% (*P* < 10^−3^) cell death (Figure 7E,F). Collectively, these results show that PRMT5 regulates the growth characteristics of MCF7, HCC1937 and BT549 cells, and that its inhibition can arrest growth and induce death of aggressive TNBC cells.

## 4 DISCUSSION

Studies on epigenetic modifications that regulate proliferative and death signalling pathways have become centre stage for therapeutic intervention. PRMT5 is now considered a key epigenetic modifying enzyme that governs major cellular process including cell proliferation and apoptosis. We have recently reported that elevated levels of PRMT5 up‐regulates WNT/β‐CATENIN proliferative signalling through transcriptional repression of pathway antagonists, *AXIN2* and *WIF1*, in three different types of non‐Hodgkin’s lymphoma cells.^37^ In the current study, we show that PRMT5 levels are up‐regulated in different types of breast cancer cell lines compared with normal human mammary epithelial cells. Our investigation revealed that PRMT5 promotes growth of transformed breast epithelial cells *in vitro* by promoting WNT/β‐CATENIN signalling via suppression of expression of WNT antagonists, DKK1 and DKK3, thereby leading to enhanced expression of WNT/β‐CATENIN target genes including *c‐MYC*, *CYCLIN D1* and *SURVIVIN*. More specifically, our experiments show that PRMT5 inhibition using the specific inhibitor, CMP5, in TNBC cell lines reduces expression of WNT/β‐CATENIN target genes, *CYCLIN D1* and *SURVIVIN*, and induces expression of pathway antagonists, DKK1 and DKK3. Our findings also show significant reduction in cell proliferation, migration and invasion in response to PRMT5 inhibition. These changes in the growth characteristics of breast cancer cells are also accompanied by induced cell death.

PRMT5 is associated with abnormal proliferation of breast cancer cells, and it was shown that PRMT5 promotes growth of MCF7 cells by increasing expression of the translation factor, eIF4E.^23^ In another study, elevated levels of PRMT5 were shown to reduce the tumour‐suppressor activity of PDCD4 in primary breast tumours by methylating its N‐terminal arginine residues, resulting in reduced patient survival.^24^ A more recent study indicated that PRMT5 levels are up‐regulated in MCF‐7, MDA‐MB‐231 and MCF‐10A cell lines as well as in clinical samples of ductal carcinoma.^25^ In line with these findings, our investigation showed that PRMT5 levels are up‐regulated in MCF7, HCC1937 and BT549 cells compared to normal human mammary epithelial cells (Figure 1). Dysregulation of WNT/β‐CATENIN signalling in breast cancer serves as a major oncogenic driver, and its up‐regulation has been implicated in many aggressive and invasive breast cancer types and correlates with acquired drug resistance.^46^, ^47^, ^48^ Aberrant activation of WNT/β‐CATENIN signalling leads to enhanced expression of downstream target genes. Our study showed that levels of WNT/β‐CATENIN target genes *CYCLIN D1*, *c‐MYC* and *SURVIVIN* are elevated in breast cancer cells (Figure 1).

Epigenetic silencing of WNT/β‐CATENIN antagonists is frequently observed in breast cancer, and reduced expression of *APC*, *CDH1*, *SFRP1* and *SFRP2* due to promoter hypermethylation has been reported in many breast tumours.^49^, ^50^ More strikingly, *WIF1* promoter hypermethylation has been frequently observed in breast cancer cells, and also serves as an early trigger in the development of hereditary breast cancers.^51^, ^52^ A previous study indicated that WIF1 expression is significantly repressed in breast cancer stem cells, which in turn promotes their self‐renewal.^53^ *AXIN1* mRNA levels were also found to be repressed in MDA‐MB‐231 cells and clinical samples.^54^ Furthermore, *DKK1* and *DKK3* promoter hypermethylation have been documented in many primary breast cancer tumours.^36^, ^55^ We have examined expression of a panel of WNT/β‐CATENIN pathway antagonists in MCF7, HCC1937 and BT549 cells, and found that expression of DKK1 and DKK3 is significantly reduced in TNBC cells (Figure 1). In light of our findings, which showed that PRMT5 is overexpressed in three different breast tumour cell lines, we checked if it was involved in inducing transcriptional repression of *DKK1* and *DKK3*. ChIP analysis showed that PRMT5 binds to the promoter region of WNT antagonists, *DKK1* and *DKK3*, and induces symmetric methylation of H3R8 and H4R3, thereby causing their transcriptional repression in HCC1937 and BT549 cells. However, this was not the case for PRMT5 recruitment to the *DKK1* promoter in MCF7 cells, where there were detectable DKK1 mRNA and protein expression (Figures 1 and 2). Lack of PRMT5 recruitment in MCF7 cells could be due to the absence of a PRMT5‐dependent transcription factor that negatively controls *DKK1* gene expression. This possibility is supported by findings, which showed that knock down of CSN5, an enzymatic component of the COP9 signalosome (CSN) that is overexpressed in many cancers, results in transcriptional derepression of *DKK1* in colorectal cancer cells.^56^ Therefore, it is possible that CSN5 stabilizes the PRMT5‐dependent transcriptional repressor in HCC1937 and BT549 cells; however, its levels might be reduced in MCF7 cells. As a result, the PRMT5‐dependent transcriptional repressor level would decline, which in turn would lead to lack of PRMT5 recruitment and increased expression of *DKK1* in MCF7 cells.

To evaluate the contribution of PRMT5 to transcriptional regulation of *DKK1* and *DKK3*, we inhibited its activity and monitored expression of both WNT/β‐CATENIN antagonists. We found that PRMT5 inhibition brings about *DKK1* and *DKK3* transcriptional derepression, which correlates with reduced expression of WNT/β‐CATENIN target genes *CYCLIN D1* and *SURVIVIN* in TNBCs (Figures 3 and 4). However, *c‐MYC* showed enhanced expression in response to PRMT5 inhibition. A prior study by Evan *et al*. (1992) showed that when expressed in the absence of proliferation signals, c‐MYC induces cell death.^57^ In agreement with this result, a later study by Murphy *et al*. (2008) showed that distinct c‐MYC expression levels regulate proliferation and apoptosis.^58^ While low levels of c‐MYC protein promote cell proliferation, its elevated expression activates the ARF/p53 tumour‐suppressor pathway, which leads to cell death.^58^ In our study, we found that PRMT5 inhibition reduces viability of breast cancer cells and induces their death (Figures 6 and 7). Therefore, it is conceivable that CMP5 treatment induces TNBC cell death through decreased expression of CYCLIN D1 and SURVIVIN, and enhanced c‐MYC expression. Further studies focusing on the mechanism by which c‐MYC induction is achieved are needed.

We have shown that PRMT5 epigenetically induces transcriptional repression of *DKK1* and *DKK3* in HCC1937 and BT549 cell lines, and that its inhibition only restores expression of both WNT/β‐CATENIN antagonists in BT549 cells. CMP5 treatment did not induce transcriptional derepression of *DKK1* and *DKK3* in HCC1937 cells, indicating that there are other epigenetic modifications involved in their regulation (Figure 3B). The fact that PRMT5 is known to induce transcriptional repression through interaction with DNMT3A,^40^ we thought that both PRMT5 and DNMT3A might be playing a similar role in silencing *DKK1* and *DKK3* in HCC1937 cells. However, treatment of HCC1937 cells with the DNA methyltransferase inhibitor, 5‐Azacytidine either alone or in combination with CMP5 did not trigger *DKK1* and *DKK3* derepression more than 1.6‐ to 1.7‐fold (Figure 3D). PRMT5 is also known to associate with histone deacetylases 2 and 3 in large multisubunit transcriptional repressor complexes.^12^, ^20^, ^59^ Therefore, we reasoned that reduced expression of *DKK1* and *DKK3* could be due to enhanced hypoacetylation of *DKK1* and *DKK3* promoter histones in HCC1937 cells. Treatment of HCC1937 cells with TSA alone or in combination with CMP5 did not induce *DKK1* and *DKK3* transcriptional derepression (data not shown). However, combinatorial treatment of HCC1937 cells with CMP5, 5‐Aza and TSA resulted in increased transcriptional derepression of *DKK1* and *DKK3*, indicating that histone lysine deacetylation, histone arginine methylation and DNA methylation work in concert to epigenetically silence DKK1 and DKK3 (Figure 3E).

ChIP assays showed that PRMT5 is enriched along with its epigenetic marks on the promoters of *DKK1* and *DKK3* in TNBC cell lines, and that its inhibition with CMP5 results in its decreased binding (Figure 5). Our previous work showed that CMP5‐mediated inhibition of PRMT5 leads to its decreased recruitment to target gene promoters, which is accompanied by reduced symmetric methylation of H3R8 and H4R3.^12^, ^37^ In agreement with these findings, our results show a similar trend of decreased PRMT5 recruitment and enrichment of its epigenetic marks in the promoter regions of *DKK1* and *DKK3* in both HCC1937 and BT549 cells. Therefore, it appears that the PRMT5 catalytic activity is required for its recruitment to target promoters.

Metastasis is a common characteristics of all aggressive breast tumours and contributes to poor prognosis and increased mortality.^45^ A previous study by Dey et al. (2013) showed that enhanced activation of WNT/β‐CATENIN signalling is positively correlated with increased metastatic potential and overall worse prognosis of breast cancer patients, highlighting the role of WNT/β‐CATENIN activation in breast cancer metastasis.^60^ Furthermore, inhibition of WNT/β‐CATENIN activation has been shown to reduce proliferation and migration of BT549 cells, and induce their death in vitro.^61^ As our study showed that PRMT5 inhibition reduces WNT/β‐CATENIN signalling in transformed breast epithelial cells, we examined the effect of PRMT5 inhibition on migration and invasion of MCF7, HCC1937 and BT549 cells. Our results showed significant reduction migration and invasion of these cells in response to PRMT5 inhibition (Figure 7A‐D). Our results also showed that PRMT5 inhibition using CMP5 LC_50_ concentration induced death of TNBCs as measured by Annexin V/PI staining (Figure 7E,F).

Our findings show that PRMT5 levels are up‐regulated in breast cancer cell lines, and that PRMT5 is involved in epigenetic silencing of a different set of WNT antagonists compared with lymphoma cells. In aggressive lymphomas, PRMT5 epigenetically inhibits expression of WNT antagonists, AXIN2 and WIF1.^37^ AXIN2 serves as a scaffolding protein in the cytosolic destruction complex, which induces β‐CATENIN degradation,^62^ whereas, WIF1 inactivates WNT/‐CATENIN signalling by directly binding to WNT ligand and inhibiting its interaction with the FZD receptor.^63^, ^64^ In TNBC cells, PRMT5 suppresses expression of DKK1, which antagonizes WNT/‐CATENIN signalling by binding to its cognate membrane receptor, Kremen2, and promoting cell surface removal of LRP5/6 by endocytosis, thereby preventing dimerization of WNT‐bound FZD with LRP5/6 co‐receptor.^65^, ^66^ DKK3 is also epigenetically suppressed by PRMT5; however, the mechanism by which DKK3 down‐regulates WNT signalling remains unclear.^55^ Regardless, it appears that PRMT5 targets various WNT/‐CATENIN pathway antagonists that operate at different levels, highlighting the complexity and diversity of its mechanism of action in the different cell types. The net outcome of PRMT5‐induced epigenetic silencing is to promote growth and survival of cancer cell, which renders its ideal for therapeutic intervention, because its selective inhibition is marked by decreased expression of pro‐survival proteins, *CYCLIN D1* and *SURVIVIN*. These molecular changes trigger reduced proliferation, migration and invasion, and increased cell death, which are all desired attributes for killing tumour cells.

## 5 CONCLUSION

Protein arginine methyltransferase 5 (PRMT5) activity is dysregulated in many aggressive cancers and its enhanced levels are associated with increased tumour growth and survival. The current study was focused on investigating the role of PRMT5 in promoting breast tumorigenesis. The results of the study showed that PRMT5 is overexpressed in breast cancer cell lines of varying aggressiveness, and that it promotes their growth by activating WNT/β‐CATENIN proliferative signalling through epigenetic silencing of pathway antagonists, *DKK1* and *DKK3*, leading to enhanced expression of *c‐MYC*, *CYCLIN D1* and *SURVIVIN*. Through chromatin immunoprecipitation (ChIP) studies, it was confirmed that PRMT5 binds to the promoter region of WNT antagonists, *DKK1* and *DKK3*, and induces symmetric methylation of H3R8 and H4R3 histones. Furthermore, PRMT5 inhibition using a specific small molecule inhibitor, compound 5 (CMP5), reduced PRMT5 recruitment as well as methylation of H3R8 and H4R3 histones in the promoter regions of *DKK1* and *DKK3*, which consequently results in reduced expression *CYCLIN D1* and *SURVIVIN*. In addition, CMP5 treatment either alone or in combination with 5‐Azacytidine and Trichostatin A restored expression of *DKK1* and *DKK3* in TNBCs. PRMT5 inhibition also altered the growth characteristics of TNBC cells including proliferation, migration and invasion, and induced their death.

## CONFLICT OF INTEREST

The authors declare that they have no conflict of interest with the contents of this article.

## Author Contribution

**Harshita shailesh:** Formal analysis (lead); Methodology (lead); Software (lead); Validation (supporting); Writing‐original draft (lead); Writing‐review & editing (supporting). **Siveen Kodappully Sivaraman:** Formal analysis (supporting); Investigation (supporting); Resources (supporting). **Saïd Sif :** Conceptualization (lead); Data curation (lead); Formal analysis (lead); Funding acquisition (lead); Investigation (lead); Project administration (lead); Resources (lead); Supervision (lead); Writing‐review & editing (lead).

## Data Availability

The data that support the findings of this study are available on request from the corresponding author. The data are not publicly available due to privacy or ethical restrictions.
